# A new twist on peer review

**DOI:** 10.7554/eLife.36545

**Published:** 2018-06-26

**Authors:** Mark Patterson, Randy Schekman

**Affiliations:** 1 eLifeCambridgeUnited Kingdom

**Keywords:** scientific publishing, peer review, research assessment

## Abstract

eLife is conducting a trial in which authors will decide how to respond to the issues raised during peer review.

The form of peer review used by eLife differs from that used by most other journals in a number of ways: for example, editors and reviewers discuss their comments before reaching a decision on a submission, and authors are sent a decision letter that consolidates these comments and outlines what authors have to do to have their manuscript accepted (King, 2017). Multiple rounds of review and revision are discouraged, and the decision letter and author response are published with the article. This month we are launching a trial to test the feasibility of an even more radical form of peer review -- an approach in which the authors will control the decision about publication and how they respond to the comments made by peer reviewers (Stern and O'Shea, 2018). The essential idea is that once an editor has invited a manuscript for full peer review, the journal is committed to publishing the work along with the reviewer reports, the decision letter, and the author response. Potential benefits of the new approach include a reduced emphasis on journal brands in research evaluation, a rebalancing of the relationship between authors, editors and reviewers, and greater efficiencies in journal publishing.

The trial process will be offered to all authors at the 'initial submission' stage until 300 have opted in. The first stage of the new process will be the same as our existing editorial process: each initial submission will be assessed by an eLife Senior Editor, usually in consultation with one or more other editors, to identify work of the highest scientific standards and potential significance. The authors of these papers will be invited to make a full submission that can be sent to external referees for peer review. Currently around one third of initial submissions go on to be peer reviewed, and roughly half of these go on to be accepted for publication. The crucial difference to our current process is that, as mentioned above, the decision to send a manuscript to external referees for peer review will be tantamount to accepting it for publication.

Under this new approach, once all the reviews have been completed, the editor and reviewers will discuss the manuscript and their comments on it, as happens now, and the editor will draft a decision letter that lists all the points that the authors must address in their response. However, the authors can decide how they respond to these points. For example, they might decide to perform additional experimental work, they might decide to adjust the claims being made in the article, they might respond to certain points in their rebuttal letter, or they might do all of the above. And if serious flaws in the work are revealed during peer review, the authors might decide to withdraw the paper. But in the end, the final choice to publish or not will be made by the authors. All articles will also have to comply with our Journal Policies; those that do not will be returned to their authors to address the concerns.

Once a revision is received, the editor, in consultation with the reviewers if necessary, will assess how the authors have responded to the issues raised during peer review and decide between the following three options: all the issues have been addressed; minor issues remain unresolved; or major issues remain unresolved. The article will then be published, with the editor's assessment appearing in a prominent position at the end of the abstract. The reviewer reports, the decision letter and the author response will also be published. Articles published as part of the trial will be designated Research Communications, to distinguish them from articles that have undergone the usual eLife process. Data on the outcomes of the trial will be collected and we will share our findings over the coming months. More details about the trial are available in this blogpost.

The decision to send a manuscript to external referees for peer review will be tantamount to accepting it for publication

We see several potential benefits to this new approach. The first relates to how it could shift the function of a selective journal, such as eLife, in the context of research evaluation. Rather than the journal name being used as a proxy for the possible quality of an article, the journal becomes a venue for the critical and transparent evaluation of work that is judged to be making important claims for a field. With articles being accompanied by the detailed thoughts of the referees, the authors’ responses to them, and the editor's assessment of the author response, those hoping to use or evaluate the work reported in an article will have much more information at their disposal.

The second potential benefit concerns the relationship between authors and reviewers. By removing the gatekeeping role of reviewers, the peer review process can focus on how the work can be strengthened. Reviewers will know that it is very likely that their comments will be published and they will have an opportunity to gain recognition for well-crafted and thoughtful advice. And authors will be incentivized to offer the most robust and careful response because the editor's assessment of their response will be available to all readers. In this way the process builds on eLife’s existing efforts to shift the editorial process towards a constructive dialog among peers, and aligns with our broader mission to foster a more collaborative and transparent scientific culture.

A third benefit is related to efficiency. Many selective journals reject a large fraction of articles after peer review, although most of these articles go on to be published after further review in other journals. If the gate-keeping function of peer review is removed and selective journals become venues for the high-quality evaluation of selected work, they would be able to publish more of the content that is submitted to them. Such a change, if broadly adopted, would save a huge amount of time and resources for authors, editors and reviewers by, for example, cutting down on 'reviewer experiments' and reducing the burden on referees caused by manuscripts being reviewed multiple times as they 'bounce' from one journal to another.

We cannot predict how authors, editors and reviewers will behave in the trial process, given that we are changing a fundamental part of what they normally do. There will almost certainly be unintended and unexpected consequences, both good and bad, and we will discuss these when we report back on the outcome of the trial. However, given that our aim is to explore how selective journals and peer review can evolve in order to support science more effectively, we are convinced that this trial is worth conducting.
